# Supplementary material for: Club cells employ regeneration mechanisms during lung tumorigenesis
Source: Nat Commun. 2022 Aug 5;13:4557. doi: 10.1038/s41467-022-32052-2 (PMC9356049; doi:10.1038/s41467-022-32052-2)
Supplement: Supplementary file 1 — Supplementary information [file 41467_2022_32052_MOESM1_ESM.pdf]

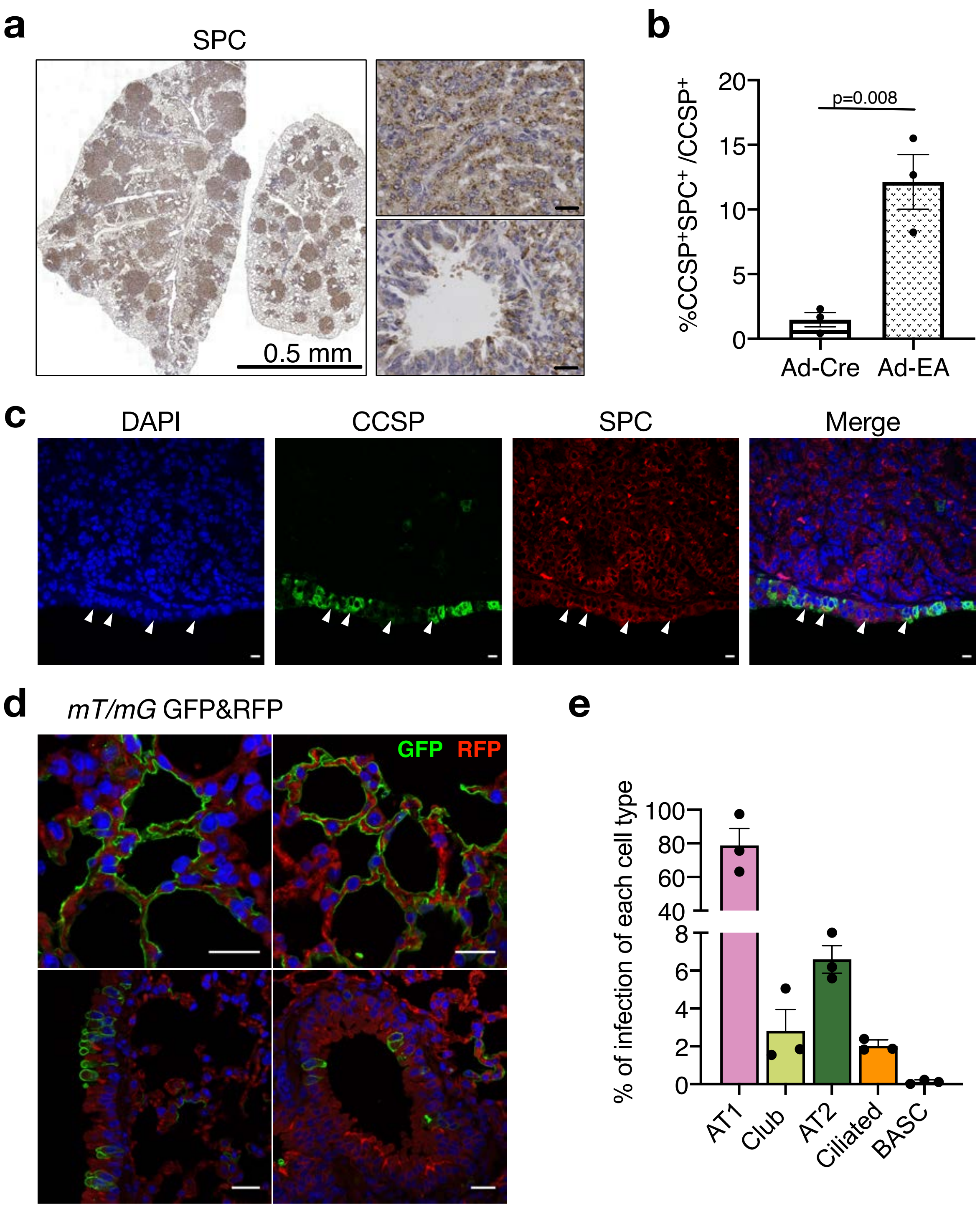

Supplementary Figure 1: Identification of adenovirus-infected cell types

a. SPC staining of a representative *Eml4-Alk* tumour bearing lung showing that all tumours are positive for SPC. Scale bars 0.5mm and 100mm. A minimum of 6 independent animals were analysed.

b. Percentage of $CCSP^{+}SPC^{+}$ double-positive cells in the $CCSP^{+}$ population in control mice (n=3, Ad-Cre) and in mice with *Eml4-Alk* induced tumours (n=3, Ad-EA). Unpaired two-tailed t test with no correction was used to calculate the p value. Data are presented as mean values +/- SEM.

c. Double-positive $CCSP^{+}SPC^{+}$ cells are found in the bronchioles during tumorigenesis. Arrowheads show double-positive cells. Antibodies are indicated. Scale bars: 10mm. A minimum of 6 independent animals were analysed.

d. GFP and RFP immunofluorescent staining on lung sections from *mT/mG* mice after infection with Ad-Cre (alveolar region, upper images; bronchiolar region, bottom images). Scale bars 20mm. A minimum of 6 independent animals were analysed. e) Percentage of each cell type infected after Ad-Cre installation in *mT/mG* mice. n=3. SEM is plotted.

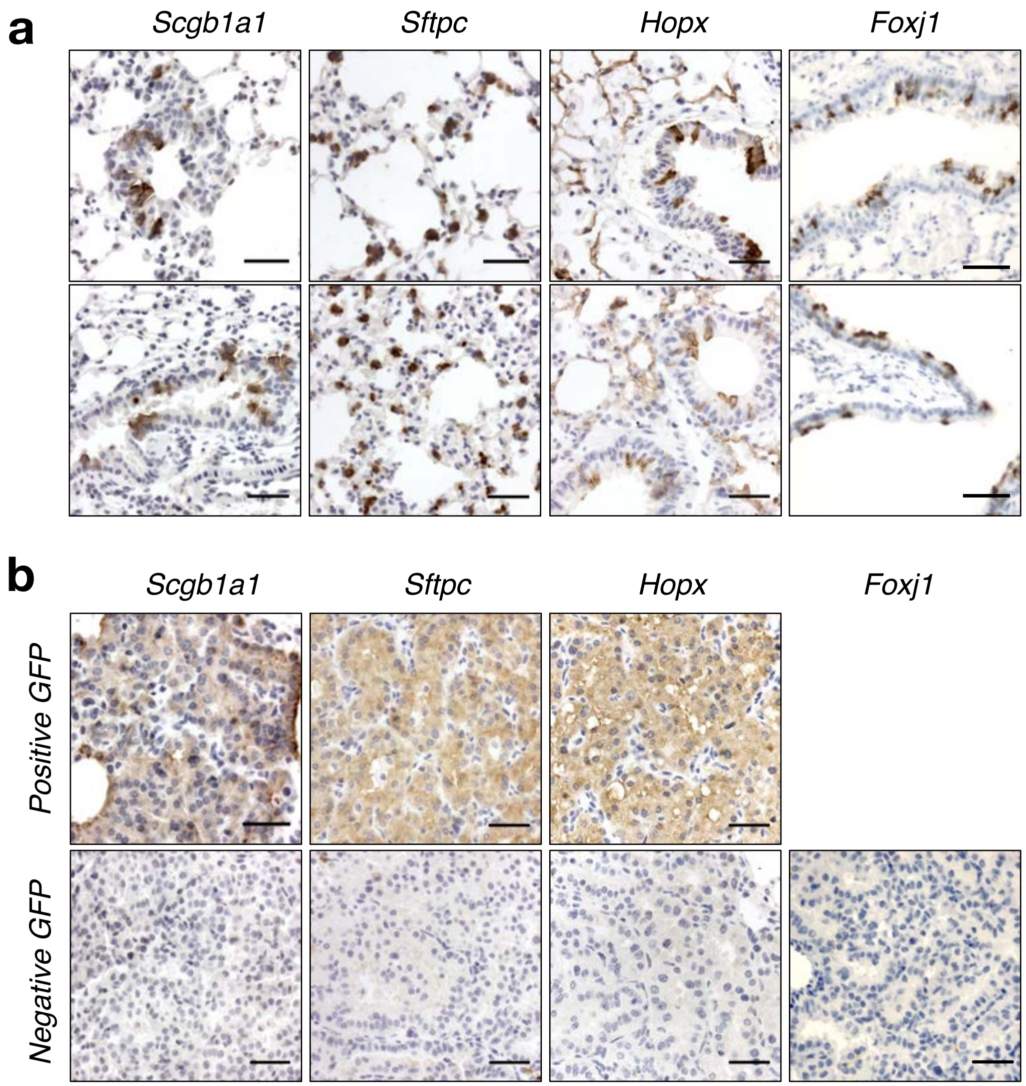

Supplementary Figure 2: GFP immunohistochemical staining on lineage tracing tissue sections

a. GFP immunohistochemical staining on lung sections from lineage-tracing mice as indicated. Scale bar 100mm. A minimum of 6 independent animals were analysed.

b. GFP immunohistochemical staining on lung tumours originated in the indicated lineage-tracing mice showing positive tumours in the upper panel and negative tumours in the lower panel. Scale bar 100µm. A minimum of 6 independent animals were analysed.

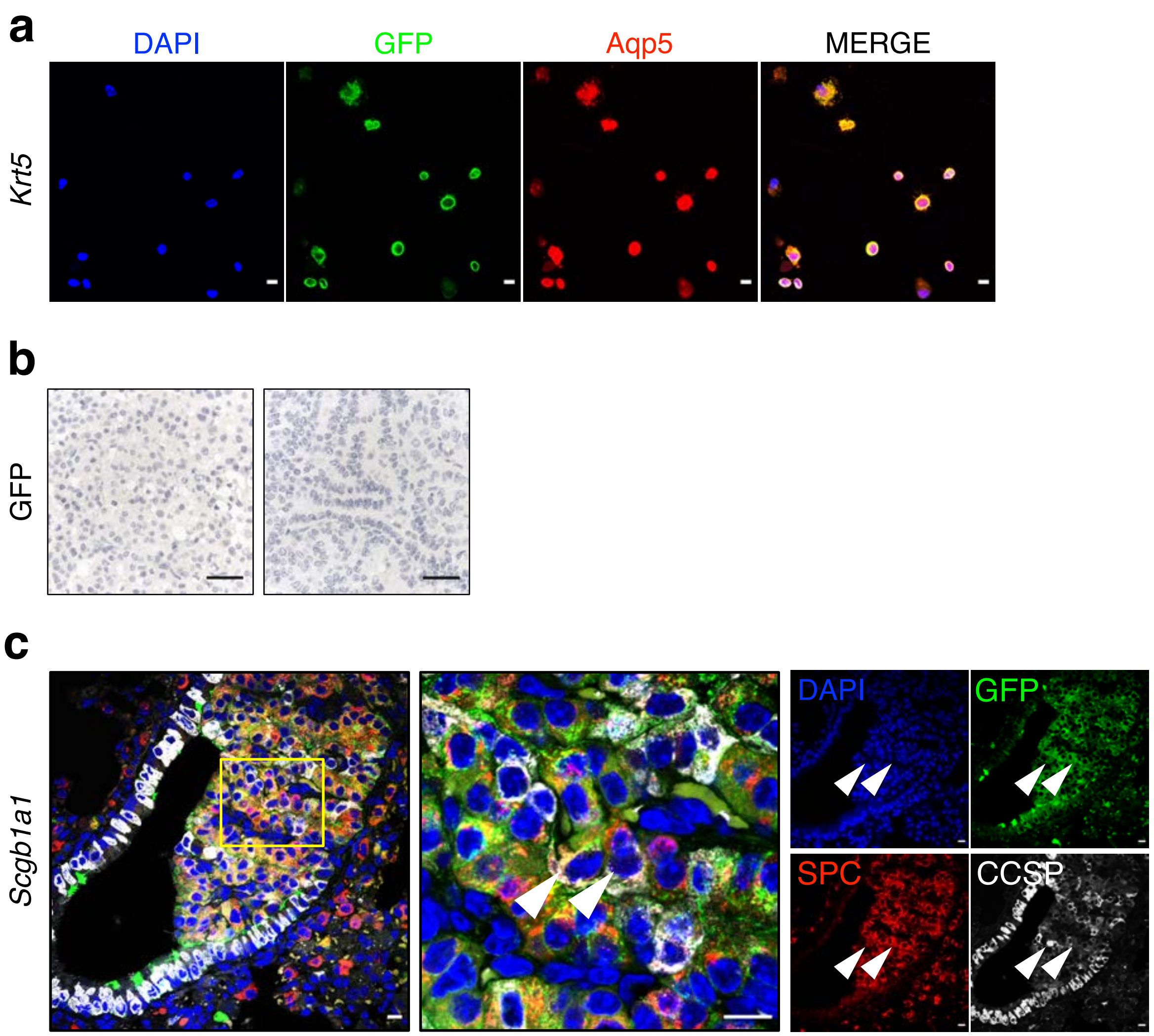

Supplementary Figure 3: Characterization of the lineage-tracing models

a. GFP and Aquaporin 5 (AQP5) immunofluorescent staining on FACS-sorted GFP$^+$ cells from *Krt5* mice. Overly and single-channel images are sequentially presented. Scale bars: 5µm. A minimum of 6 independent animals were analysed.

b. GFP immunohistochemical staining on the tumours from *Krt5* mice. Scale bars: 50µm. A minimum of 6 independent animals were analysed.

c. GFP, SPC and CCSP immunofluorescent staining of early lesions originated in *Scgb1a1* lineage-tracing mice. Magnification of the highlighted area is shown on the right and arrowheads indicate Club cells under lineage switch. Scale bar: 10µm. A minimum of 6 independent animals were analysed.

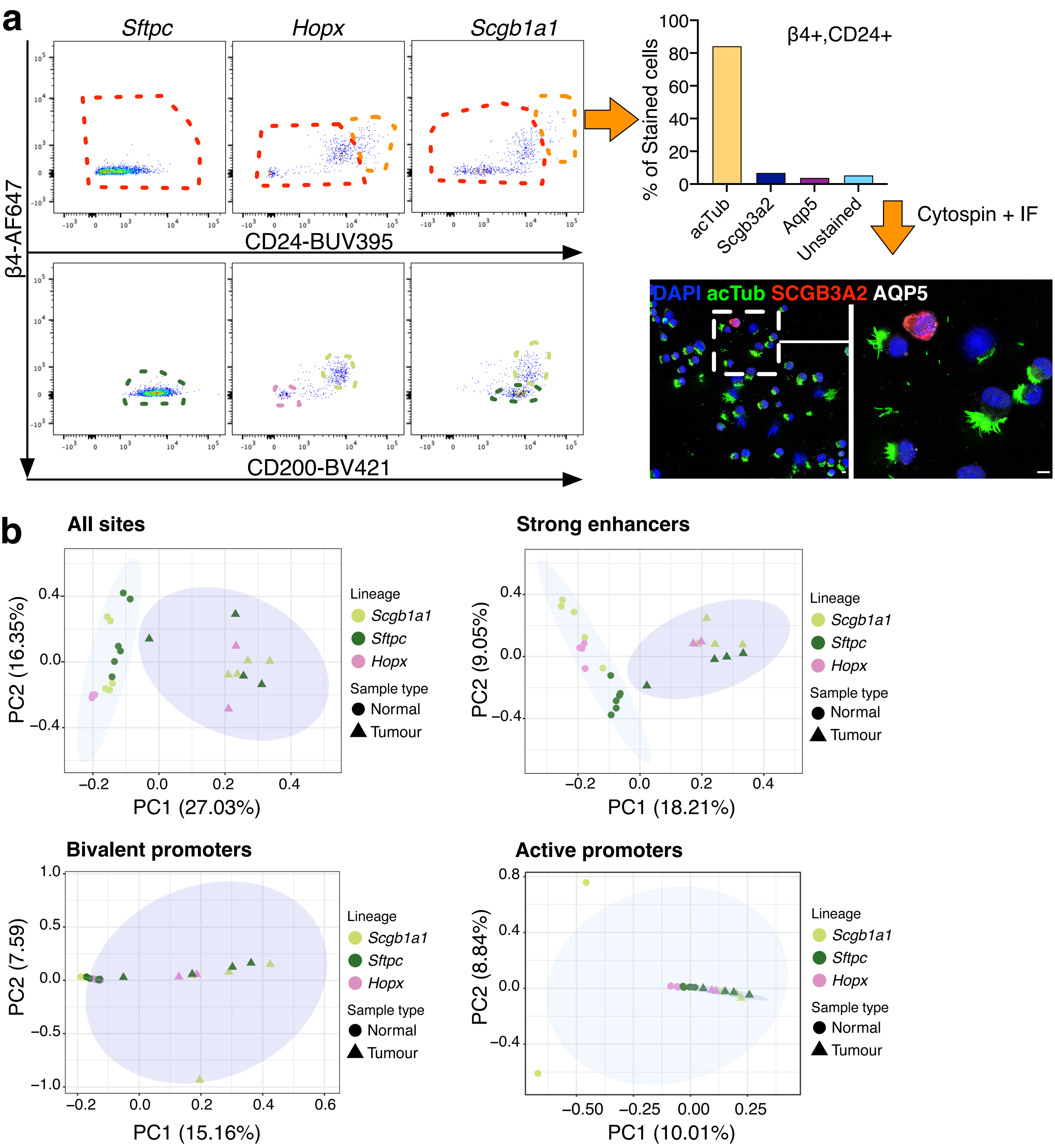

Supplementary Figure 4: Tagmentation-based whole-genome bisulfite sequencing sorting and PCA plots

a. Sorting strategy of the isolated GFP^{+} cells in the indicated lineage-tracing models for tagmentation-based whole-genome bisulfite sequencing (TWGBS). Dashed lines indicate different cell types that compose the sorted GFP^{+} population: orange, Ciliated cells (β4^{+}, CD24^{+}); red, non-ciliated cells (β4^{+}, CD24^{-}). Out of the non-ciliated cells (red gate), there are AT2 cells (dark green; β4^{-}, CD200^{-}), Club cells (light green; β4^{+}, CD200^{+}), and AT1 cells (pink; β4^{-}, CD200^{-}). Further demonstration of the Ciliated cell population is provided through immunofluorescent staining of the sorted β4^{+}, CD24^{+} population using the indicated cell type specific markers (acTub for Ciliated cells, SCGB3A2 for Club cells and AQP5 for AT1 cells). A total of 266 cells from 3 views was used to generate the bar plot.

b. Principal component analysis plot of DNA methylation. CpG sites were selected in different ways: top variable sites from all regions (all sites), as well as sites overlapping with strong enhancers (En-Sd, En-Sp), bivalent promoters (Pr-B), or active promoters (Pr-A). The functional regions were defined based on ChromHMM tracks from postnatal 0-day old mouse lung (ENCODE, ENCSR538YJF).

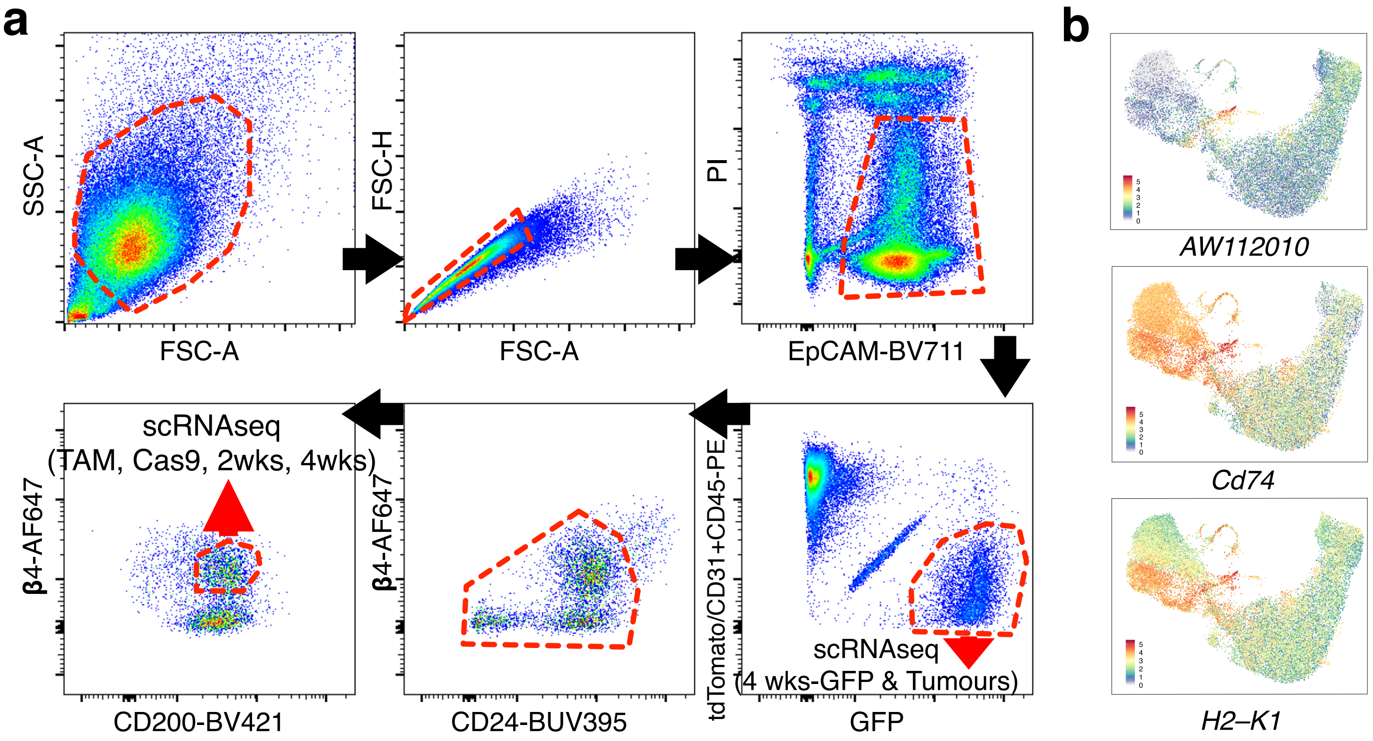

Supplementary Figure 5: Single-cell RNA sequencing and expression of marker genes

a. Sorting strategy to obtain GFP labelled Club cells in the *Scgb1a1* line for scRNA-seq. Dashed lines indicate the gates applied to select the subpopulations from the parental cells. Arrows indicate the cell populations that were sequenced.

b. UMAP embedding with cells coloured by the expression levels of *AW112010*, *Cd74,* and *H2-K1*. X- and y-axes represent the first and second UMAP dimensions (UMAP_1 and UMAP_2), respectively.

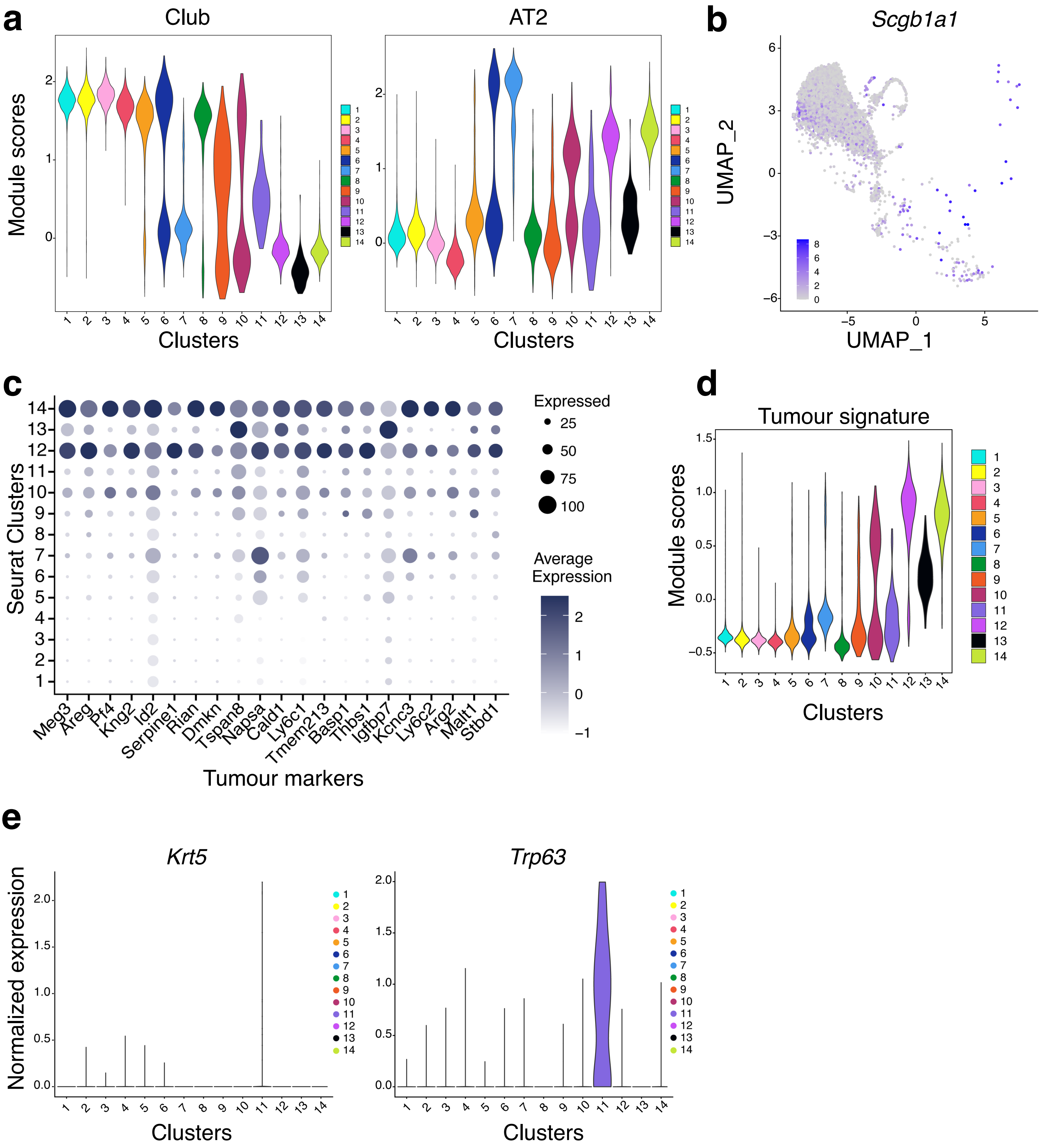

Supplementary Figure 6: Single cell RNA sequencing clusters and activity programs

a. Violin plots showing the expression module score of Club (left) and AT2 (right) signatures by the identified clusters. The signatures were used as described by Strunz et al. (Strunz et al., 2020).

b. UMAP embedding with cells from the tumour sample coloured by the expression levels of *Scgb1a1*. X- and y-axes represent the first and second UMAP dimensions (UMAP_1 and UMAP_2), respectively.

c. Dot plot showing the high expression of tumour genes mainly in clusters 12 and 14. The size of the dots represents the percentage of cells in the cluster that expresses the gene, while the intensity of the colour shows the average expression of the gene.

d. Violin plot indicating the module score of the tumour signature by the identified clusters (signature composed by the genes in **c**.

e. Violin plot depicting the normalized expression of *Krt5* and *Trp63* by cluster indicating elevated expression in cluster 11.

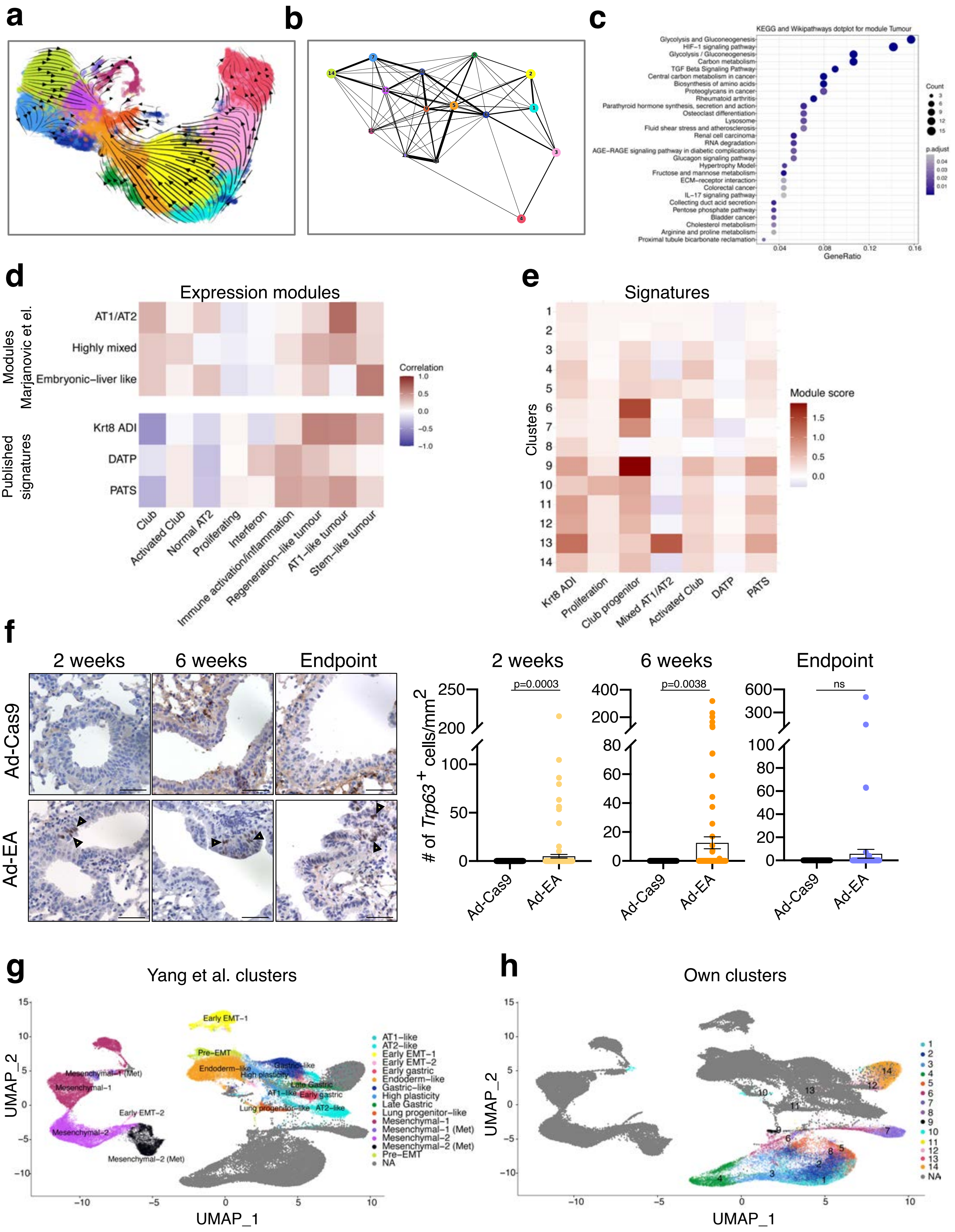

Supplementary Figure 7: Trajectories of club cells, correlation with Kras tumour cells and validation

a. RNA velocity plot projected to the UMAP indicating the directional progression of transcriptional states of the clusters.

b. Partition-based graph abstraction (PAGA) analysis projected to the identified clusters. Cluster nodes connected with weighted edges (width of the connections).

c. Pathway enrichment analysis based on KEGG and WikiPathways for the regeneration-like tumour activity module, identified by cNMF. The size of the dots shows the number of genes among the top 200 genes. The colour represents level of significance. X-axis depicts the gene ratio between the number of genes in the pathway and the number of genes among the selected ones.

d. Heatmap of the correlation between the identified activity programs and the programs identified by Marjanovic et al. or previously published signatures (Choi et al., 2020; Kobayashi et al., 2020; Strunz et al., 2020) (Supplementary Data 4). In the upper part, the colours represent the correlation between the z-score unit gene expression of the gene activity programs calculated using cNMF, while the lower part shows the correlation of module scores calculated using the top 10 genes of the activity program or the genes of the published signatures.

e. Module scores of previously identified signatures by cluster (Choi et al., 2020; Kobayashi et al., 2020; Strunz et al., 2020).

f. Immunohistochemistry of Trp63 in Ad-Cas9 controls and representative Eml4-Alk lungs at different time points after adenoviral instillation. Scale bar 20µm. Right: quantification of Trp63 positive cells/mm2 of bronchi of the animals. Each dot represents a bronchus. Two-tailed Mann-Whitney test. Data presented as mean values +/- SEM. n= 3 mice per group were analysed except Ad-Cas9 2 weeks (n=4) and Ad-Cas9 6 endpoint (n=2).

g. UMAP of the integration result of the presented study and the data from Yang et al., 2022. Integration was done using the Seurat 4 R package with reciprocal PCA based on the top 4000 genes. Cells are coloured based on the clusters of Yang et al. Grey cells represent cells in our study.

h. UMAP of the integration result of the presented study and the data from Yang et al., 2022. Integration was done using the Seurat 4 R package with reciprocal PCA based on the top 4000 genes. Cells are coloured based on the clusters of the present study. Grey cells represent cells from Yang et al., 2022.

Supplementary Table 1. Percentage (%) of infected cells after Ad-cre

Number and percentage of different epithelial cells in the lung after infection of mT/mG mice with Ad-Cre

mouse id	AT1/GFP	AT2/GFP	club/GFP	ciliated/GFP	BASC/GFP
TG26268	97,43	8,48	1,85	1,83	0,12
TG26269	63,23	5,59	5,06	2,40	0,01
TG25950	75,61	6,19	1,55	1,88	0,23

Mean	**78,76**	**6,76**	**2,82**	**2,03**	**0,12**
SD	14,14	1,25	1,59	0,26	0,09

Supplementary Table 2. Labelling rate of lineage tracing mice.

Labelling rate of the different lung cell types in the specified linage tracing mouse models. For each mouse line and cell type the following are included: the number of positive cells for the cell type marker; the number of double positive cells (GFP and cell type marker); the percentage of GFP labeled cells out of the cell type entire population.

		AT2 cells			Club cells			Ciliated cells		
	Sample	SPC	GFP+SPC	labelling	CCSP	GFP+CCSP	labelling	acTub	GFP+acTub	labelling
Scgb1a1	GCRC504	31861	3520	11,05	123012	95783	77,86	29646	4241	14,31
	GCRC507	23334	1497	6,42	8018	3947	49,22	816	218	26,73
	GCRC514	26120	4086	15,64	40990	17430	42,52	5716	968	16,93
	GCRC517	16450	2051	12,47	166537	126740	76,10	6320	2247	35,56
	Average			**11,39**			**61,43**			**23,38**
	SD			3,32			15,75			8,42

		AT2 cells		
	Sample	SPC	GFP+SPC	labelling
Sftpc	DTS118	72521	42914	59,17
	DTS120	35361	17883	50,57
	GCRS506	53655	18815	35,07
	GCRS508	37494	13865	36,98
	Average			**45,45**
	SD			9,93

		AT1 cells			Club cells			Ciliated cells		
	Sample	PDPN	GFP+PDPN	labelling	CCSP	GFP+CCSP	labelling	acTub	GFP+acTub	labelling
Hopx	GCRH425	14576179	2186621	15,00	67417	21807	32,35	17109	4201	24,56
	GCRH426	12481118	1955346	15,67	22142	8639	39,02	5015	848	16,90
	GCRH433	13583189	1397243	10,29	57924	20703	35,74	34860	5611	16,10
	Average			**13,65**			**35,70**			**19,18**
	SD			2,39			2,72			3,81

		Ciliated cells			Club cells		
	Sample	acTub	GFP+acTub	labelling	CCSP	GFP+CCSP	labelling
Foxj1	GCRF9	7368	2956	40,12	7272	96	1,32
	GCRF10	11729,09	1919	16,36	7758	88	1,14
	Average			**28,24**			**1,23**
	SD			11,88			0,09

Supplementary Table 3. Percentage of GFP positive tumors in each of the mouse strains.

Percentage of GFP tumors in each mouse model

	mouse id	GFP+ tumours	total tumours	% GFP
Scgb1a1	GCRC21	29	63	46,03
	GCRC79	10	27	37,04
	GCRC31	20	41	48,78
			mean	**43,95**
			sd	5,02

	mouse id	GFP+ tumours	total tumours	% GFP
Sftpc	GCRS39	2	32	6,25
	GCRS145	3	22	13,64
	GCRS125	18	93	19,35
	GCRS101	11	40	27,50
	GCRS12	0	7	0,00
	GCRS40	1	20	5,00
			mean	**11,96**
			sd	9,34

	mouse id	GFP+ tumours	total tumours	% GFP
Hopx	CRH83	8	44	18,18
	GCRH417	14	38	36,84
	GCRH419	1	35	2,86
			mean	**19,29**
			sd	13,90

	mouse id	GFP+ tumours	total tumours	% GFP
Foxj1	GCRF17	0	56	0,00
	GCRF102	0	27	0,00
	GCRF111	0	14	0,00
	GCRF119	0	16	0,00
			mean	**0,00**

	mouse id	GFP+ tumours	total tumours	% GFP
Krt5	GCRK33	0	14	0
	CRK84	0	56	0
	GCRK28	0	17	0
	GCRK340	0	43	0
			mean	**0,00**

Supplementary Table 4. Cell type composition in each of the time points.
Cell type composition of each cell type based on the results of single-cell gene expression

	TAM	Cas9	2 wk-1	2 wk-2	4 wk-1	4 wk-GFP	Tumour
Chia 1 positive cells	16,00	15,90	9,27	13,70	10,50	5,55	2,30
AT1/AT2-like tumour	0,00	0,00	0,00	0,03	0,00	0,07	1,40
Activated AT2	0,03	0,94	0,07	2,68	2,69	19,50	0,44
AT2 cells	1,30	2,79	0,89	3,37	6,75	61,00	5,96
Activated Club	1,33	1,61	0,19	11,80	12,70	0,07	0,04
Club cells	80,90	76,60	88,20	60,80	57,10	7,29	2,01
H2-K1 high club-like progenitors	0,50	0,69	0,52	2,68	6,19	1,13	1,54
Pre-tumour stage	0,03	0,03	0,09	0,09	0,88	0,03	0,28
Proliferating cells	0,83	0,85	0,61	4,22	1,81	0,64	4,07
Tumour cells	0,20	0,51	0,17	0,58	1,33	4,10	82,00
