# Supplementary material for: Club cells employ regeneration mechanisms during lung tumorigenesis
Source: Nat Commun. 2022 Aug 5;13:4557. doi: 10.1038/s41467-022-32052-2 (PMC9356049; doi:10.1038/s41467-022-32052-2)
Supplement: Supplementary file 3 — Reporting Summary [file 41467_2022_32052_MOESM3_ESM.pdf]

nature portfolio

Corresponding author(s): Rocio Sotillo

Last updated by author(s): Jun 30, 2022

Reporting Summary

Nature Portfolio wishes to improve the reproducibility of the work that we publish. This form provides structure for consistency and transparency in reporting. For further information on Nature Portfolio policies, see our Editorial Policies and the Editorial Policy Checklist.

Statistics

For all statistical analyses, confirm that the following items are present in the figure legend, table legend, main text, or Methods section.

n/a	Confirmed	
☐	☒	The exact sample size (n) for each experimental group/condition, given as a discrete number and unit of measurement
☐	☒	A statement on whether measurements were taken from distinct samples or whether the same sample was measured repeatedly
☐	☒	The statistical test(s) used AND whether they are one- or two-sided<br>*Only common tests should be described solely by name; describe more complex techniques in the Methods section.*
☐	☒	A description of all covariates tested
☐	☒	A description of any assumptions or corrections, such as tests of normality and adjustment for multiple comparisons
☐	☒	A full description of the statistical parameters including central tendency (e.g. means) or other basic estimates (e.g. regression coefficient) AND variation (e.g. standard deviation) or associated estimates of uncertainty (e.g. confidence intervals)
☐	☒	For null hypothesis testing, the test statistic (e.g. F, t, r) with confidence intervals, effect sizes, degrees of freedom and P value noted<br>*Give P values as exact values whenever suitable.*
☒	☐	For Bayesian analysis, information on the choice of priors and Markov chain Monte Carlo settings
☐	☒	For hierarchical and complex designs, identification of the appropriate level for tests and full reporting of outcomes
☒	☐	Estimates of effect sizes (e.g. Cohen's d, Pearson's r), indicating how they were calculated

Our web collection on statistics for biologists contains articles on many of the points above.

Software and code

Policy information about availability of computer code

Data collection	No software was used
Data analysis	FlowJo (Version10.1)<br>methylCtools Hovestadt et al., 2014 https://github.com/hovestadt/methylCtools<br>MethylDackel N/A https://github.com/dpryan79/MethylDackel<br>cNMF, v. 1.2 Kotliar et al., 2019 https://github.com/dylkot/cNMF<br>Homer, v. 4.11 Heinz et al., 2010 http://homer.ucsd.edu/homer/<br>Cell Ranger, v. 3.1 10X Genomics https://support.10xgenomics.com/single-cell-gene-expression/software/pipelines/latest/installation<br>Methrix R package, v. 1.4.07. Mayakonda et al., 2020 https://github.com/CompEpigen/methrix<br>bsseq R package, v. 1.26.0 Hansen et al., 2012 https://bioconductor.org/packages/release/bioc/html/bsseq.html<br>MeDeCom R package, v. 1.0.0 Lutsik et al., 2017 https://github.com/lutsik/MeDeCom<br>DSS R package, v. 2.38.0 Park and Wu, 2016 https://bioconductor.org/packages/release/bioc/html/DSS.html<br>ggplot2 R package, v. 3.3.3.9000 Wickham https://ggplot2.tidyverse.org/<br>ComplexHeatmap R package, v. 2.6.2 Gu et al., 2016 https://jokergoo.github.io/ComplexHeatmap-reference/book/<br>fgsea R package, v. 1.16.0 Korotkevich et al., 2021 https://bioconductor.org/packages/release/bioc/html/fgsea.html<br>Seurat R package, v. 3.2.2 Stuart et al., 2019 https://satijalab.org/seurat/<br>biomaRt R package, v. 2.46.3 Durinck et al., 2005; 2009 https://bioconductor.org/packages/release/bioc/html/biomaRt.html<br>Harmony R package, v. 1.0 Korsunsky et al., 2019 https://github.com/immunogenomics/harmony<br>org.Mm.eg.db R package, v. 3.12.0 N/A https://bioconductor.org/packages/release/data/annotation/html/org.Mm.eg.db.html<br>clusterProfiler R package, v. 3.18.1 Yu et al., 2012 https://guangchuangyu.github.io/software/clusterProfiler/<br>annotatr R package, v. 1.16.0 Cavalcante and Sartor, 2017 https://bioconductor.org/packages/release/bioc/html/annotatr.html<br>scvelo Python pacakge, v. 0.2.3 Bergen et al., 2020 https://scvelo.readthedocs.io/

scanpy Python pacakge, v. 1.7.2 Wolf et al., 2018 https://scanpy.readthedocs.io/en/stable/

All original code has been deposited at Github (https://github.com/tkik/Lung_CoO) and is publicly available as of the date of publication.

For manuscripts utilizing custom algorithms or software that are central to the research but not yet described in published literature, software must be made available to editors and reviewers. We strongly encourage code deposition in a community repository (e.g. GitHub). See the Nature Portfolio guidelines for submitting code & software for further information.

Data

Policy information about availability of data

All manuscripts must include a data availability statement. This statement should provide the following information, where applicable:

- Accession codes, unique identifiers, or web links for publicly available datasets
- A description of any restrictions on data availability
- For clinical datasets or third party data, please ensure that the statement adheres to our policy

Single-cell RNA-seq and DNA methylation data have been deposited at GEO (GEO: GSE176186) and are publicly available as of the date of publication. Accession numbers are listed in the key resources table. All original code has been deposited at Github (https://github.com/tkik/Lung_CoO) and is publicly available as of the date of publication

Field-specific reporting

Please select the one below that is the best fit for your research. If you are not sure, read the appropriate sections before making your selection.

[x] Life sciences [ ] Behavioural & social sciences [ ] Ecological, evolutionary & environmental sciences

For a reference copy of the document with all sections, see nature.com/documents/nr-reporting-summary-flat.pdf

Life sciences study design

All studies must disclose on these points even when the disclosure is negative.

Sample size	The number of mice used for the studies was approved by Baden-Wurttemberg, Germany (animal license No. G185-17, G265-19). At least 3 animals per group was used for statistical purposes. For methylome analysis: 25 samples . For scRNAseq, 7 mice.
Data exclusions	For the single-cell RNAseq data, cells were filtered for quality control. After the initial analysis, a small cluster of Ciliated cells marked by high expression of Foxj1 was identified and excluded. For methylome, two samples were removed due to quality issues.
Replication	The results reported in this manuscript were reproducible in independent experiments with the exception of the methylome and scRNA seq, that was performed once.
Randomization	Mice were randomized to different timepoint groups without selection.
Blinding	Investigators were not blinded to group allocation since this was not required in the experiments.

Reporting for specific materials, systems and methods

We require information from authors about some types of materials, experimental systems and methods used in many studies. Here, indicate whether each material, system or method listed is relevant to your study. If you are not sure if a list item applies to your research, read the appropriate section before selecting a response.

Materials & experimental systems

n/a	Involved in the study	
[ ]	[x]	Antibodies
[x]	[ ]	Eukaryotic cell lines
[x]	[ ]	Palaeontology and archaeology
[ ]	[x]	Animals and other organisms
[x]	[ ]	Human research participants
[x]	[ ]	Clinical data
[x]	[ ]	Dual use research of concern

Methods

n/a	Involved in the study	
[x]	[ ]	ChIP-seq
[ ]	[x]	Flow cytometry
[x]	[ ]	MRI-based neuroimaging

Antibodies

Antibodies used	Microscopy: ProSP-C (Millipore, AB3786), CC10 (Santa Cruz, SC-9772), acetylated tubulin (Sigma, T7451), Cytokeratin 5 (Abcam, ab53121), Podoplanin (Abcam, ab11936), GFP (Cell Signalling Technology, 2956S), GFP (Abcam, ab5450), RFP (Rockland

600-401-379), Aquaporin 5 (Abcam, ab78486), Scgb3a2 (R&D systems, AF3465), GFP (Cell Signalling Technology, 2956S), CCSP (Millipore, 07-623), p63 (Sigma, ab735), Lactotransferin (Sigma, 07-685), Alexa 488 donkey anti-rabbit IgG (Thermofisher, A21206), Alexa 488 donkey anti-goat IgG (Abcam, ab150129), Alexa Fluor 488 Donkey anti-mouse IgG (Thermofisher Scientific, A-21202), Alexa 568 donkey anti-rabbit IgG (Thermofisher, A10042), Alexa 568 donkey anti-goat IgG (Thermofisher, A11057) and Alexa 568 donkey anti-mouse IgG (Abcam, ab175700), Alexa Fluor 647 Donkey anti-mouse IgG (Thermofisher Scientific, A-31571), Alexa Fluor 647 Goat Anti-hamster IgG (Thermofisher Scientific, A21451).

FACs: DAPI (Thermofisher Scientific, 62248), Rat PE Anti-mouse CD45 (BioLegend, 103106), Rat PE Anti-mouse CD31 (BioLegend,102408), Rat BV711 anti-mouse CD326 (Ep-CAM) (Biolegend, 118233), Rat BUV395 Anti-CD24 (BD Biosciences, 744471), Rat Alexa Fluor 647 Anti-CD104 (Biolegend, 123608) and Rat BV421 Anti-CD200 (BD Biosciences, 565547).

Validation

All antibodies have been validated by the manufacturer and by multiple citations for reactivity against mouse. For FACS, compensation beads have been additionally used.

Animals and other organisms

Policy information about studies involving animals; ARRIVE guidelines recommended for reporting animal research

Laboratory animals

In this manuscript 6-12-week-old female and male mice were used. mT/mG mice were used in C57BL/6 and FVB mixed background. Hopx-CreERT2 mice were in mixed background between 129 and FVB and the Krt5-CreERT2 line in C57BL/6 and SJL and FVB mixed background. Sftpc-CreER-rtTA, Scgb1a1-CreERT and Foxj1-CreERT were used in mixed C57BL/6 and FVB background.

Wild animals

The study did not involve wild animals

Field-collected samples

The study did not involve samples collected from the field.

Ethics oversight

All animal experiments had been approved by the local veterinary authorities and from the Regierungsprä sidium Karlsruhe, Baden-Wurttemberg, Germany (animal license No. G185-17, G265-19).

Note that full information on the approval of the study protocol must also be provided in the manuscript.

Flow Cytometry

Plots

Confirm that:

- [x] The axis labels state the marker and fluorochrome used (e.g. CD4-FITC).
- [x] The axis scales are clearly visible. Include numbers along axes only for bottom left plot of group (a 'group' is an analysis of identical markers).
- [x] All plots are contour plots with outliers or pseudocolor plots.
- [ ] A numerical value for number of cells or percentage (with statistics) is provided.

Methodology

Sample preparation

All cells were extracted immediately after mice were sacrificed. Healthy lung and tumours were dissociated into single cells using the lung dissociation kit (Miltenyi Biotech, 130-095-927) in a gentleMACS Octo Dissociator (Miltenyi Biotech, 130-095-937). After depletion of red blood cells with the red blood cell lysis buffer (Sigma, R7757), tumours samples were incubated with tumour cell isolation kit (Miltenyi Biotech, 130-110-187), while normal samples were incubated with CD31 and CD45 microbeads (Miltenyi Biotech, 130-110-187). Cells from the flow-through were collected and DAPI was added as a viability marker. Cells were then sorted in a BD FACSAria cell sorter.

Instrument

BD FACSAria cell sorter

Software

FlowJo (Version10.1)

Cell population abundance

Describe the abundance of the relevant cell populations within post-sort fractions, providing details on the purity of the samples and how it was determined.

Gating strategy

FSC/SSC was used to gate cells.

- [x] Tick this box to confirm that a figure exemplifying the gating strategy is provided in the Supplementary Information.
